# The economic cost of fatal workplace accidents in Sweden – A methodology for long-term decision analysis

**DOI:** 10.3233/WOR-211120

**Published:** 2023-05-16

**Authors:** Mahmoud Rezagholi

**Affiliations:** Department of Business and Economic Studies, Division of Economics, University of Gävle, SE-801 76, Gävle, Sweden E-mail: madrei@hig.se

**Keywords:** Safety and security, human capital, labour productivity, societal perspective, effective working years

## Abstract

**BACKGROUND:**

The few studies attempting to estimate costs of fatal accidents at workplaces suffer from poor or obscure applied methodologies. As the costs are often limited for the exposed company/industry in the short run, economic decisions about investments to improve the safety and security of workplaces are moreover not made at the societal level nor within an appropriate time frame. In a social economic decision, the total potential productivity lost over time due to a fatal accident is considered regardless of who pays what compensation to the families involved.

**OBJECTIVE:**

This study introduces a methodology appropriate for making long-term economic decisions at the societal level to prevent accidents in Swedish workplaces.

**METHODS:**

The introduced methodology, which is based on the human capital approach, is used to assess potential productivity losses associated with the accidents.

**RESULTS:**

The empirical findings show that, over the period 2008–2019, Swedish society could have gained more than 8.5 billion Swedish crowns by preventing accidents at Swedish workplaces.

**CONCLUSION:**

The objective achieved as the economic cost of fatal workplace accidents assessed from a long-term societal perspective. Effective preventive measures in the workplace make thus an incredible contribution to society in the form of increased national income, sustainable welfare and economic development.

## 1 Introduction

Safety and security at work constitute an important factor in the production of goods and services in every country. Safe and secure workplaces improve the performance of human capital and thus organizational production and national income. The buildings and equipment in a workplace must consequently undergo regular maintenance and the workers undergo regular education and training according to an effective safety program. Failures and deviations from a safe and secure workplace can lead to an increase in accidents, which is costly for society, for workers and their families, and for firms, as well as for social insurance systems and government. A safe and secure workplace is thus an important pre-condition for a satisfying work life, health, social welfare, and sustainable economic development.

Workplace accidents are a serious global problem with major consequences for society. Fatalities, permanent disability and work impairment, along with a high social cost, are the worst of these consequences. According to the Global Burden of Disease Study 2015 (GBD 2015), work-related mortality accounted for 5% of the total global toll, with fatal accidents making up 13.7% of that work-related mortality. We also know that the number of fatal occupational accidents in 2014 was 8% higher than the corresponding figure for 2010. The consequences of non-fatal accidents that lead to absenteeism and presenteeism are less clear, however, since these can be underestimated and are not reported at all by many countries [1]. Researchers in the field have mainly addressed the *causes* of industrial workplace accidents and focused less on the *costs* that highlight the need to invest in preventive measures.

### 1.1 Causes of workplace accidents

Many of the studies have focused on work-environment and socio-cultural causes of accidents in the workplace, identifying a number of factors in- and outside the workplace that carry a high risk of accidents. A lack of safety programs has, for example, been cited as the cause of chemistry accidents in laboratories including, in one case, the death of a UCLA (University of California, Los Angeles) researcher [2].

Alcohol and drugs are known general factors that significantly increase the risk of accidents at work. A study of a large railway transport company in Portugal showed that random and unexpected testing for alcohol and drugs (A&D) in the workplace had a statistically significant preventive effect on safety and reduction of individuals’ accident risk, where the fraction of prevented accidents was 59% for on-board train workers, 72% for those working near trains, and 85% for white-collar workers [3].

Studies have also looked at internal risk factors of workplace accidents in different industries. For instance, a study of the cause of an increase in fatal accidents in Malaysia’s construction industry found unsafe methods, the unique nature of the industry, and job-site conditions to be the top three causes, with working at high elevations, incorrect work procedures, and structure failures as sub-causes [4]. However, the high number of fatal accidents and the causes in the Malaysian construction industry are also likely due to the lack of a safety culture and non-compliance with the country’s Occupational Safety and Health Act [5]. The impacts of human factors in fatal workplace accidents have also been studied in Brazil (for the period 2007 to 2011), where three organizational factors were found to be important determining risk factors – non-compliance with safety standards, deficiencies in assessing the work risk, and supervision failures [6]. Outsourcing has also been found to increase the risk of accidents [7] and, in a study of Mexican workers, socio-demographic factors (age, sex and occupation) as well as work environment and workplace conditions were all associated with fatal accidents in the workplace [8].

Studies of the causes of workplace accidents often lack an in-depth analysis of the conclusive risk factors. However, the work environment is a multidimensional reality [9, 10], and the research area should identify which of the psychosocial, ergonomic and physical risk factors at workplaces in each industry are most decisive in causing the accidents in question. This has been done, for example, for lost work time, work impairments, and labour productivity [11–16]. Crucial and suspected risk factors of workplace accidents include conflicts, violent behaviour, alienation, discrimination, stress, job insecurity and dissatisfaction, job demands, long work shifts, instability, handling heavy objects, and working in poorly lit environments. Some of the suspected work environment risk factors have also recently been studied, with one systematic review having shown extended working hours to be significantly associated with an increased risk of accidents [17], and another study showing higher suicide mortality rates among Korean workers [18]. Non-standard employment such as through a temporary agency and seasonal work was also associated with increased mortalities among workers [19], and the severity of workplace accidents despite an underreporting of severe injuries [20]. Further, emotional contagion in the form of anger and joy has also been associated with greater and fewer cognitive failures, respectively, which are linked to higher rates of subsequent workplace accidents [21]. Workplace accidents caused by workplace failures have also been shown to be associated with psychological distress [22]. In addition, a correlation has been shown between self-reported occupational accidents and employment conditions, job demands and workplace justice [23].

### 1.2 The cost sources of accidents in the workplace

The costs identified in the workplace accident literature consist of direct administrative and medical costs, indirect income reduction and loss of productivity, and intangible human costs [24]. There are at present not many scientific research and commercial reports that attempt to estimate the costs of workplace accidents, and those that do exist are impeded by unclear cost sources and accident outcomes at the same time as using either poor or obscure methodologies [25]. Capturing these economic costs, however, is a must for making rational decisions regarding any investment to prevent accidents in the workplace. Certain, rational and effective decision-making in this context also requires an appropriate cost assessment methodology. The misreporting of these costs can lead to a knowledge gap or suggesting insufficient preventive measures.

There are, however, some studies in the relevant literature that have estimated the cost of workplace accidents. The Liberty Mutual Workplace Safety Index (WSI), for example, ranks the top ten causes of serious workplace injuries according to their direct cost to U.S. businesses. The 2019 WSI lists overexertion involving outside sources, same-level falls, and being struck by an object or equipment as the three most costly non-fatal injuries reported, at 13.11, 10.38 and 5.22 billion USD, respectively, while striking an object or equipment was the least costly, at 1.15 billion USD. With respect to non-fatal injuries, the WSI further states the construction sector to be the most costly industry, while the leisure and hospitality sector was the least costly [26].

In the case of accidents with fatal outcomes, the costs can be substantial if we include the indirect economic cost to society as a whole [25]. The total cost of all work-related injuries may also be much higher if we take into account long-latency disabilities, the causes of which are difficult to establish [27]. There is thus a potential to underreport causes (i.e. risk factors of workplace accidents) as well as to underestimate the cost of diseases. The European Agency for Safety and Health at Work conducted a review of studies evaluating the cost of work-related injuries, finding that a variety of methods and approaches were used to assess this cost [25].

There are challenges surrounding the approaches and methodologies used to measure the cost of workplace accidents before making decisions regarding safety and security in the workplace. The social costs associated with workplace accidents are usually estimated using one of two well-known cost models: *accounting* costs, and *economic* costs based on the human capital approach (HCA) [28]. The accounting cost estimation model includes all registered or measurable costs, no matter who in society pays. The main costs measured in this model include: administrative costs, social security and social insurance payments, healthcare costs, and friction costs for the affected firms [29]. A firm’s circumstances and status in the market can also come into crisis after the occurrence of work-related accidents, leading to an additional cost. An indirect economic or opportunity cost model based on HCA focuses instead on potential societal income losses [30, 31]. There are also intangible human costs associated with workplace accidents, such as physical pain and suffering, and the impact on an accident victim’s family [25]. These costs can dramatically decrease the quality of life and life expectancy of workplace accident survivors with permanent or temporary disabilities, further diminishing social welfare and sustainability. As mentioned, the human costs are intangible, which means they are difficult to identify, to measure and, especially, to value in monetary terms. Because the existing intangible costs associated with workplace accidents can be decisive, however, there is an indirect economic model – the willingness to pay (WTP) model – for valuation of decreased quality of life following non-fatal accidents at work [32, 33].

When conducting a cost assessment for economic decisions at the societal level, we should also add in the indirect costs of lost government tax revenues and employer friction costs to the direct accounting costs [30]. When it comes to workplace accidents, however, this cost assessment model contains a big uncertainty in that no one knows how many accidents initially listed as “non-fatal” may change to fatal or how many of the affected workers may become permanently disabled during the friction period. Both workplace accident outcomes and the length of the friction period are unknown. In practice, the cost model only entails a short-term assessment of social costs associated with workplace accidents. Conversely, the indirect economic cost assessment model takes a long-term societal perspective and does not need to add any other costs. However, in the human capital approach the definition of labour productivity contains both market productivity at work and non-market productivity at home, and optimally also at social enterprises engaged in during leisure time [30].

### 1.3 Modelling economic evaluation of preventive measures

Because studies of workplace accidents often lack cost data about safety programs and accident-prevention measures, the current literature offers no models for decision-making and economic evaluations of alternative safety intervention programs, though models have been developed for work-related disorders [30, 31, 34–36]. Researchers in the field prefer to highlight the importance of minimizing the risk and costs of accidents, rather than focusing on the cost of implementing different measures to improve safety and security in the workplace.

### 1.4 The importance of societal perspective in reports of workplace accidents and accident cost assessments

Scientific research in this area should include a societal perspective since the whole society pays for workplace accidents that occur in an industry. All potential costs incurred by all stakeholders, irrespective of who pays, should be considered. In order to do this, researchers need complete information about workplace accidents that have occurred – about the productivity of the affected workers at work and at home, in addition to how their families and surrounding community, government, and social organizations and enterprises are affected. Researchers and decision-makers also need information on the spaces, equipment and tasks with a high risk for accidents in the exposed industry [6] in order to suggest possible preventive measures and to assess their costs.

The socio-economic consequences of occupational accidents are characterized by long time horizons and a broad and diverse number of social areas [25]. The costs of workplace accidents, especially those with fatal outcomes, are thus allocated across time and across society through different stakeholders at different levels. Policy-makers should therefore know that, even if they frequently require costs, measures undertaken to prevent accidents also generate utility over time via the potential human (non-economic) and economic cost savings – for both industry and society as a whole – of preventing accidents [7]. The human capital approach addresses the potential societal income lost through workplace accidents – an economic cost to society that could be saved by preventing the accidents in the first place.

### 1.5 Fatal workplace accidents in Sweden

According to the Swedish Work Environment Authority, workplace accidents with fatal outcomes in Sweden for 2010–2019 had three top causes (AV/ISA). The first cause was lost control – primarily of vehicles, but also of machinery, tools, animals or other objects. The second top cause of fatal workplace accidents in the country was bursts, explosions and landslides or sliding objects, and the third falling or slipping. These three causes were responsible for 52%, 17% and 14%, respectively, of all fatal accidents in workplaces during the period noted.

When it comes the industries with occupational accidents leading to death – the construction, transport and agricultural sectors topped the list, representing 57% of all fatal workplace accidents in Sweden for the same period, 2010–2019. At the opposite end of the scale – hotels, restaurants, and the information and communication sectors were the safest workplaces in Sweden during the period.

In the period looked at in the current study using registered data and information by Swedish Work Environment Authority (2008–2019), 73 people who died in workplace accidents in Sweden (an average of 6 workers per year) were foreign workers working for foreign enterprises. The information for these workers is therefore registered in other countries and not included in our cost assessment model here.

### 1.6 Objective

The purpose of this study was to introduce an appropriate economic cost assessment model that can be used prior to rational decision-making at the macro level regarding prevention of accidents at Swedish workplaces. The economic cost could therefore be assessed from a long-term societal perspective.

## 2 Materials and methods

Data and information about fatal workplace accidents in Sweden between 2008–2019 was collected from yearly reports issued by the Swedish Work Environment Authority. These reports contain demographic and occupational data on workers involved in fatal accidents during the year, such as age, sex, profession and industry, as well as a brief description of the events that led to the fatalities. Based on the registered data and information on age and profession, other complementary data such as the salaries of the workers and work productivity indices were collected from the National Mediation Office. The opportunity cost of not preventing workplace accidents was estimated based on productivity losses in terms of income lost to society due to fatal accidents at Swedish workplaces 2008–2019.

The economic cost assessments were based on the following criteria and assumptions:

1) Only socio-economic costs that could be expressed/valued in monetary terms were included. Human costs such as consequences for affected family and friends, and loss of productivity in non-profit areas, such as social enterprises, were considered “intangible costs”.
2) The current inflation rate was used to discount the annual opportunity cost.
3) The percentage annual salary increase for both skilled and unskilled labour was assumed to be unchanged, and was the figure used as the index for labour productivity increase.
4) The effective age of retirement for farmers is 74, and for workers in other sectors it is 67 (the current general retirement age in Sweden).

The economic costs associated with fatal workplace accidents occur at very different points in time and cannot be compared directly. A long-run economic analysis of workplace accidents should thus consider the effect of *time*. That is, allow for differential timing in assessing related economic costs. The costs have therefore been adjusted for inflation to estimate their *present value* [30]. According to Swedish monetary policy, the given inflation rate is around 2%. Discounting enables us to perform a rational comparative analysis of the yearly cost and effective decision-making about prevention of accidents at workplaces.

### 2.1 The economic cost assessment model

The indirect economic cost (EC) of each fatal workplace accident is estimated based on the marginal revenue product of labour per month (*MRP*_*L*/*M*_), which indicates the work function’s potential contribution to the industry’s output per month:

(1) $${\mathrm{MRP}}_{L/M}=k\cdot w\cdot {\left(1+r\right)}^{n},$$

where *k* is a coefficient for the societal cost of an employee and indicates the potential gain to society from the function; *w* is the national wage; *r* is the inflation-adjusted annual wage increase; and *n* is the number of years remaining after the fatal accident before the deceased worker would have reached the effective age of retirement.

The deceased worker *i* would have increased national income during his/her effective working years (number of months *j* remaining until retirement) by:

(2) $${\mathrm{EC}}_{i}=\sum_{j}^{1}{\mathrm{MRP}}_{L/M},$$

and also would have increased social welfare through non-market (home) productivity by:

(3) $${\mathrm{HPL}}_{i}=\sum_{j}^{1}100\cdot {\bar{w}}_{h},$$

where *HPL*_*i*_ stands for home productivity losses; 100 is the estimated number of hours per month that each individual would have devoted to work in the home, according to Statistics Sweden (SCB); and ${\bar{w}}_{h}$ is the estimated (unpaid) average hourly wage for different work at home based on market prices.

The total economic cost of a fatal workplace accident (*TEC*_*i*_), including productivity losses at work and at home, is thus estimated as the sum of *EC*_*i*_ and *HPL*_*i*_:

(4) $$E({\mathrm{TEC}}_{i})={\mathrm{EC}}_{i}+{\mathrm{HPL}}_{i}$$

### 2.2 Adjusting for differential timing

As the economic costs of fatal workplace accidents are allocated across a period of several years that varies for each deceased worker, a decision to improve workplace safety and prevent accidents must consider the present value of the cost, estimated as:

(5) $$\begin{array}{cc} E(\mathrm{TEC})= & \sum_{n}^{1}{\mathrm{TEC}}_{i}={\mathrm{TEC}}_{i(n=0)}+\frac{\mathrm{TEC}}{\left(1+ɛ\right)}\\ & +\frac{\mathrm{TEC}}{{\left(1+ɛ\right)}^{2}}+...+\frac{\mathrm{TEC}}{{\left(1+ɛ\right)}^{n}}, \end{array}$$

where *ɛ* stands for the market interest rate.

## 3 Results

Using the above-described methodologies, the characteristics and estimated economic costs of fatal workplace accidents in Sweden for the years 2008–2019 are presented in the following tables. All costs are given in SEK and rounded to integers. At the time of writing (6 May 2021), the rates of exchange for EUR and USD to SEK were 10.16 and 8.42, respectively.

As shown in Table 1, during the years 2008–2019 Sweden lost a total of 543 workers (an average of 45.25 per year) and 10,863 effective working years (an average of 905.25 per year) due to fatal workplace accidents.

**Table 1:** Characteristics of the study participants (*N* = 35)

	Year	Number of deaths	Lost working years	Over working age
	2008	68	1641	5 over 67; 4 over 74
	2009	41	891	2 over 67; 1 at 74
	2010	54	1205	3 over 67; 1 over 74
	2011	58	1072	4 over 67; 3 over 74
	2012	45	854	2 over and 3 at 67; 1 over 74
	2013	35	549	4 over 67; 1 at 74
	2014	41	765	2 over and 1 at 67
	2015	34	632	1 at 67; 3 over 74
	2016	37	713	3 over 67; 1 over 74
	2017	44	787	7 over and 1 at 67; 5 over 74
	2018	50	1086	3 over 67; 1 over 74
	2019	36	668	2 over and 1 at 67; 1 over 74
Total		**543**	**10,863**	**44** **≥** **67; 22** **≥** **74**
Average		**45.25**	**905.25**	**3.67** **≥** **67; 1.83** **≥** **74**

As expected, the cost to Swedish society of fatal workplace accidents during the period 2008–2019 was very high (See Table 2) – a huge loss of potential productivity valued at more than 8.5 billion SEK (on average more than 712 million/year and over 16.5 million per accident). The economic costs for Swedish society indicate the high societal value of safety and security at work.

**Table 2:** The socio-economic costs (i.e. lost value to society in monetary terms) of fatal workplace accidents in Sweden

	Year	Annual cost	Average cost per accident	Average cost per month
	2008	1,216,589,290	19,009,208	101,382,441
	2009	686,620,304	16,746,837	57,218,359
	2010	916,036,451	17,616,086	76,336,371
	2011	787 500,651	14,583,345	65,625,054
	2012	644,062,109	14,978,189	53,671,842
	2013	424,842,246	12,998,063	35,403,520
	2014	583,307,069	14,956,592	48,608,922
	2015	490,626,128	15,826,649	40,885,511
	2016	605,948,641	17,822,019	50,495,720
	2017	700,552,898	18,933,862	58,379,408
	2018	900,469,076	18,759,772	75,039,090
	2019	593,781,008	17,464,147	49,481,751
Total cost		**8,550,335,871**
Average cost		**712,527,989**	**16,641,231**	**59,377,332**

In analysing economic decisions regarding alternative safety programs in workplaces, the costs of these programs should be compared with their expected societal value, including the rate of the programs’ ability to prevent fatal workplace accidents.

## 4 Discussion

The indirect economic cost of fatal accidents at societal level in Sweden for 2008–2019 is estimated. In the case of direct costs, while the most crucial cost is that of funeral costs, the payment of which can only be deferred by preventing fatal accidents, the direct costs were not considered from a long-term societal perspective. In addition, as economic costs of fatal workplace accidents have not been empirically estimated in the collected relevant studies, the empirical result obtained in this study cannot be evaluated and compared over time and by countries.

It is worth noting, however, that not all workplace accidents are fatal, and not all work-related mortalities are the obvious result of accidents. There are many other causes of work-related mortality besides fatal workplace accidents, and unreported and underestimated non-fatal accidents can furthermore be much more costly [1]. If we count the costs of non-fatal accidents, the value of lost national income in Sweden each year may thus be several times greater than the cost of investing in preventing accidents in the workplace. Compared with studies of entire occupational injuries, the total direct cost of the most disabling workplace injuries to U.S. business in alone one year (2019) was 55.43 billion USD [26].

### 4.1 Internal and external causes of workplace accidents

Organizational factors in workplaces can lead to emotional reactions and stress, in turn leading to accidents at work. For instance, the ever-fluctuating and high quantity and quality demands on production made by profit-maximizing firms in the market as they battle for competitive advantage lead to high stress among workers [18, 23]. Swedish workplaces and the exposed workers cannot be isolated from Swedish society, however, so some causes and sub-causes of accidents at work are not due to the work environment but to individual, demographic and socio-cultural conditions that mainly develop in the workplace. An effective workplace safety program can thus not prevent all accidents and their related societal costs. As earlier studies have shown, human factors, safety culture and the misuse of alcohol and drugs, for example, have led to workplace accidents [3, 5, 6]. However, it is extremely difficult to determine the extent to which fatal work accidents in Sweden are caused by internal or external factors. When making economic decisions at the macro level regarding the prevention of workplace accidents, decision-makers should therefore consider all inside and outside environmental factors since non-work-related factors also affect people’s socio-economic functions and thus the organizational and social value of health and safety [8].

### 4.2 The human capital approach and its limitations in the cost assessments

The assessment of the economic cost of fatal workplace accidents was based on the concept of human capital. Introduced by Schultz (1960) and further defined by Becker (1987), the concept of human capital encompasses potential forces and capabilities embodied in humans that lead to the creation of individual and social well-being [28]. Applying the concept of human capital substantially is a useful approach when assessing lost opportunities and potential productivity from a societal perspective, as it has the potential to consider determinants such as structures of the economy and its labour market. The human capital approach upon which the economic cost of fatal accidents in Sweden is assessed, however, is salary-based and assumes that, in a perfectly competitive labour market, wages should reflect the workers’ marginal contributions to a firm’s output. The equilibrium price of labour (salary), which is determined in the labour market, is accordingly equal to the marginal revenue product of labour (MRPL) [30]. There are at least two conditions that should be met for this equality. The first is the existence of equal job opportunities, and the second the existence of the “same salary for the same job” [30, 37]. In the case of monopsony and high unemployment, a worker’s salary is less than the MRPL because of workers’ low bargaining power [30, 37]. When a market fails, salaries can also be affected by the health status of workers and can vary between genders, and ethnic and age groups [37]. It is not clear, however, that the Swedish labour market is under perfect competition and free from discrimination. When assessing the MRPL on the basis of salaries, one must thus consider the state of the country’s labour market in terms of unemployment and market failures (monopsony power and discrimination) that lead to allocative inefficiencies (deadweight losses) [37].

In addition to the assumptions in the underlying economic theories, such as constant salary increases and stable inflation of around 2% and a perfectly competitive labour market, there are other limitations in the cost assessment model:

1) With the exception of productivity losses at work and at home, it was not possible to assess non-economic human costs or other socio-economic costs. Thus, due to the existence of many intangible and non-measurable yet definite economic and non-economic costs, the cost assessment model based on the human capital approach may not over-estimate the true cost of fatal workplace accidents in Sweden. The potential social benefits of preventing accidents at Swedish workplaces were in fact larger than the average national wage that the assessment model was based on.
2) With respect to the economic cost assessment model, it was assumed that farmers and other workers work up to the age of 74 years and 67 years, respectively. However, a number of the deceased workers, e.g. truck drivers and farmers, were over these age limits when the fatal accidents occurred. Thus, it is difficult to know just how much longer some workers would have worked. In the current study, the cost of fatal accidents was not included for deceased workers over the age limits.
3) It was also assumed that the deceased workers would have continued to work in the same industry, at the same position, with the same skills and competence, and at the same work capacity as when the accidents occurred, which is not realistic for younger workers. By preventing fatal accidents at Swedish workplaces, the affected workers would have the chance to increase their skills and thereby income, leading to a sustainable technical and economic development. All of society would gain from this in terms of long-term growth in social welfare. Not knowing the potential increase to productivity is an additional source that could lead to underestimation of the cost of fatal workplace accidents in Sweden.
4) Sources of overestimation of the costs also exist, as it was assumed that the exposed workers would otherwise live through to retirement or to the end of their effective working life.

### 4.3 Prior to an economic decision to prevent accidents at work

The reports of fatal accidents published by the Swedish Work Environment Authority did not contain all of the data and information needed to make economic decisions on preventing workplace accidents in Sweden. The first step of the decision-making process for a safe and secure workplace is to identify the spaces, equipment and tasks with a high risk for workplace accidents in high-risk industries. This type of data and information is useful in order to propose suitable preventive measures. In the case of alternative measures, the second step is to assess the cost and effectiveness of the measures, whereas the cost of a unique preventive measure should be compared to its opportunity cost (i.e. the economic cost of fatal workplace accidents) or the social utility of preventing accidents at work. For the former, the approaches of cost-effectiveness analysis (CEA), cost utility analysis (CUA) and cost-minimization analysis (CMA) can be applied. For the latter, social return on investment (SROI), socio-economic cost analysis (SECA) and cost benefit analysis (CBA) can be used. A calculation of willingness to pay (WTP) can also be used prior to evaluation of any preventive measure. The areas of application for these methodologies are as follows:

1) CEA compares the cost of alternative safety programs and their social utility yield in terms of accident reduction. The key concept of *effectiveness* is defined as accident-free working days.
2) CUA compares the costs of alternative safety programs and their social utility yield in terms of quality-adjusted accident reduction. Here, the term *utility* should be redefined as accident-free working days multiplied by feeling safe at work.
3) CMA: If several feasible options for preventing accidents are put forward, with different costs and different expected risk-reduction effects, decision-makers can implement the least expensive measure or the one that minimizes the risk for accidents, or equivalency, the opportunity cost of not preventing accidents into consideration. The measure with the lowest cost per reduced risk of accident is the cost-effective choice, while still fulfilling any constraints.
4) SROI focuses on changes in value created by preventive measures, in terms of occupational safety, social welfare and sustainability, rather than financial returns.
5) SECA compares the actual investment costs of preventing accidents with potential opportunity cost, i.e. the socio-economic cost of not investing.
6) CBA compares the cost of each preventive measure with its anticipated benefits at the societal level. To accept a measure, the benefits should be larger than the cost, i.e.:
  Anticipated benefits (= economic cost of workplace accidents * preventive measure’s likelihood of preventing accidents)>the cost of prevention.
7) WTP can be used for valuation of non-market outcomes (in monetary terms) of alternative preventive measures.

All of the approaches and methodologies described here have been successfully developed and applied in social sciences as well as occupational health economics for making decisions regarding implementation of proposed programs at different levels [32–38].

### 4.4 Recommendations for future research

Occupational health economics as new developing science attempt to allocate the scarce resources to improve occupational health by providing rational decision-making models [9–11, 30, 31, 35, 36, 41]. The subject is thus in need of developing appropriate economic evaluation methodologies to assess future costs and benefits associated with alternative measures. The human capital approach used in this study is, however, a robust foundation for developing such methodologies. The economic cost of fatal accidents at Swedish workplace was assessed in this study using the approach. Economic decision-makings as well require an assessment of costs associated with different available preventive measures using the same approach. Studies of workplace accidents with an economic perspective should therefore be developed in this way while trying to model the employers’ efforts to improve the work environment [10].

## 5 Conclusions

The objective of this study achieved as the economic cost of fatal workplace accidents assessed by using an appropriate cost assessment model from a long-term societal perspective. Swedish society could have benefitted to a total of over 8.5 billion SEK by preventing the fatal workplace accidents that occurred over the period 2008–2019. Effective preventive measures in the workplace make an incredible contribution to society in the form of sustainable welfare and development.

## Ethical approval

Not applicable.

## Informed consent

Not applicable.
